# Unveiling the potential of mitochondrial dynamics as a therapeutic strategy for acute kidney injury

**DOI:** 10.3389/fcell.2023.1244313

**Published:** 2023-08-11

**Authors:** Yajie Hao, Limei Zhao, Jing Yu Zhao, Xiutao Han, Xiaoshuang Zhou

**Affiliations:** ^1^ The Fifth Clinical Medical College of Shanxi Medical University, Taiyuan, China; ^2^ The Third Clinical College, Shanxi University of Chinese Medicine, Jinzhong, Shanxi, China; ^3^ Department of Nephrology, Shanxi Provincial People’s Hospital, The Fifth Clinical Medical College of Shanxi Medical University, Shanxi Kidney Disease Institute, Taiyuan, China

**Keywords:** mitochondrial dynamics, acute kidney injury, mitochondrial fusion, mitochondrial fission, therapeutic targets

## Abstract

Acute Kidney Injury (AKI), a critical clinical syndrome, has been strongly linked to mitochondrial malfunction. Mitochondria, vital cellular organelles, play a key role in regulating cellular energy metabolism and ensuring cell survival. Impaired mitochondrial function in AKI leads to decreased energy generation, elevated oxidative stress, and the initiation of inflammatory cascades, resulting in renal tissue damage and functional impairment. Therefore, mitochondria have gained significant research attention as a potential therapeutic target for AKI. Mitochondrial dynamics, which encompass the adaptive shifts of mitochondria within cellular environments, exert significant influence on mitochondrial function. Modulating these dynamics, such as promoting mitochondrial fusion and inhibiting mitochondrial division, offers opportunities to mitigate renal injury in AKI. Consequently, elucidating the mechanisms underlying mitochondrial dynamics has gained considerable importance, providing valuable insights into mitochondrial regulation and facilitating the development of innovative therapeutic approaches for AKI. This comprehensive review aims to highlight the latest advancements in mitochondrial dynamics research, provide an exhaustive analysis of existing studies investigating the relationship between mitochondrial dynamics and acute injury, and shed light on their implications for AKI. The ultimate goal is to advance the development of more effective therapeutic interventions for managing AKI.

## 1 Introduction

Mitochondria, commonly referred to as the ‘powerhouse of the cell' play a crucial and pivotal role among the various organelles within the cellular environment. Their primary function is to convert nutrients into energy, providing the cell with essential biological power, such as ATP. In addition to energy production, mitochondria are involved in vital cellular processes, including apoptosis, mitophagy, metabolic regulation, control of cellular inflammation, and oxidative stress responses. Therefore, maintaining mitochondrial function is essential for the normal functionality of the cell (Kasai et al., 2020; Martínez-Reyes and Chandel, 2020; Marchi et al., 2023).

Mitochondria are composed of an inner and an outer membrane, with intermembrane space (IMS) between them. The inner mitochondrial membrane (IMM) contains numerous folded structures known as mitochondrial cristae. These cristae provide attachment sites for the electron transport complexes responsible for the electron transfer chain. Through a series of complex reactions, these complexes facilitate the production of ATP, the biological energy currency, via the respiratory chain process (Colina-Tenorio et al., 2020). Mitochondria exhibit a highly dynamic nature, constantly changing their shape, position, and number in response to changes in the cellular environment. This dynamic behavior, encompassing mitochondrial migration, fusion, fission, and mitophagy, is collectively referred to as "mitochondrial dynamics".

Mitochondrial migration involves the directed movement of mitochondria within the cell, facilitated by interactions with the cellular cytoskeleton. Mitophagy, on the other hand, is a cellular process responsible for the degradation and removal of damaged or aging mitochondria, thus preserving mitochondrial quality and functionality. Of particular interest are mitochondrial fusion and fission processes. Mitochondrial fusion refers to the merging of two or more mitochondria into one, facilitating the mixing of mitochondrial contents and maintaining mitochondrial integrity. Conversely, mitochondrial fission involves the division of a single mitochondrion into two or more smaller mitochondria, playing a critical role in cellular proliferation and repair. Both mitochondrial fusion and fission serve as prerequisites for other mitochondrial regulatory mechanisms, including mitophagy and mitochondrial biogenesis (Chan, 2020). These processes hold significant importance in mitochondrial physiology and have emerged as focal points of research into mitochondrial regulatory mechanisms. In recent years, researchers have extensively studied the regulatory mechanisms of mitochondrial fusion and fission, identifying numerous proteins and molecular pathways involved. For instance, dynamin-related protein 1 (Drp1) is responsible for mitochondrial fission, while optic atrophy 1 (OPA1) supports mitochondrial fusion.

The use of mitochondria as therapeutic targets has gained popularity in various disease research, including AKI. The kidney, being a high-energy consuming organ rich in mitochondria (Bhargava and Schnellmann, 2017), often experiences mitochondrial dysfunction in cases of AKI, as supported by numerous studies (Maekawa et al., 2019; van der Slikke et al., 2021; Xiao et al., 2022). Among the mitochondrial regulatory mechanisms, mitochondrial dynamics, particularly fusion, and fission, play a key role in the onset and progression of AKI. In this context, we will focus on the recent advancements in mitochondrial dynamics research, explore the relationship between mitochondrial dynamics and AKI, and examine the potential significance of targeting mitochondrial dynamics as a therapeutic strategy for AKI. This will provide a theoretical foundation for the development of new treatment approaches.

## 2 Mechanisms underpinning mitochondrial morphological alterations

### 2.1 Mitochondrial fission

Mitochondrial fission is a complex process involving the participation of numerous proteins, with the GTP hydrolase, dynamin-related protein 1 (Drp1) serving as a central player. Drp1 interacts directly or indirectly with other fission-related proteins (Pagliuso et al., 2018), making it a key component in unraveling the entire process of mitochondrial fission.

Initially, Drp1 proteins are not located on the mitochondrial surface but rather reside in the cytoplasm. This cytoplasmic localization is possibly associated with the retrograde motor protein dynein-dynactin complex (Varadi et al., 2004). To facilitate mitochondrial fission, Drp1 must be recruited from the cytoplasm to the surface of the mitochondrial membrane. This recruitment process involves complex interactions and transcriptional modifications of various proteins, which will be discussed in detail.

The structure of the Drp1 monomer can be divided into four parts: the head, neck, trunk, and foot (Kraus et al., 2021). The head contains the core of Drp1, which is the GTPase structure responsible for binding and hydrolyzing GTP. The neck region harbors the Bundle Signaling Element (BSE), while the intermediate domain between the head and neck acts as a bridge connecting the GTPase structure domain and the BSE. The trunk comprises the stalk structure domain, which includes important interfaces for self-aggregation and is involved in the formation of X-shaped dimers (Ford et al., 2011). The foot region, located at the bottom of Drp1, facilitates its interaction with various proteins (Bui and Shaw, 2013; Ugarte-Uribe et al., 2014; Stepanyants et al., 2015). This region also exerts inhibitory effects on the formation of Drp1 polymers (Francy et al., 2015).

The translocation of Drp1 from the cytoplasm to the mitochondria involves several modification processes, including phosphorylation, ubiquitination, SUMOylation, O-GlcNAcylation, and nitrosylation. Among these, phosphorylation has been extensively studied, with protein kinase A (PKA) and Ca^2+^/calmodulin-dependent protein kinase Iα (CaMKIα) being responsible for the phosphorylation and dephosphorylation of Drp1 at Ser637, respectively. Phosphorylation inhibits Drp1’s GTPase activity, while dephosphorylation activates it (Chang and Blackstone, 2007; Han et al., 2008). Activated GTPase activity has been closely associated with Drp1 mitochondrial aggregation and the promotion of mitochondrial fission (Smirnova et al., 2001; Chang and Blackstone, 2007; Cribbs and Strack, 2007; Cereghetti et al., 2008). However, recent research suggests that despite Ser637 phosphorylation, Drp1 can still be recruited to the outer mitochondrial membrane (OMM) and mediate mitochondrial fission, indicating that Ser637 phosphorylation may act as an influencing factor rather than a determinant (Yu et al., 2019b). In a research investigation concerning diabetic kidney disease, it was observed that elevated glucose levels prompt the phosphorylation of Drp1 at the Ser637 site. This, in turn, leads to the recruitment of Drp1 to mitochondria, resulting in the initiation of mitochondrial fission (Chen et al., 2020b). Due to the intricate nature of Drp1 modifications, it is hypothesized that modifications at additional sites may potentially counteract the inhibitory effect on GTPase activity resulting from Ser637 phosphorylation. Ser616 represents another critical phosphorylation site on Drp1. In contrast to Ser637, phosphorylation at Ser616 is typically viewed as an activator of Drp1, promoting its recruitment. Conversely, dephosphorylation at Ser616 hinders Drp1’s recruitment to mitochondria. A myriad of kinases are reported to phosphorylate Drp1 at Ser616, including cyclin-dependent kinase 1 (CDK1), extracellular signal-regulated kinase 1/2 (ERK1/2), Rho-associated coiled-coil kinase (ROCK), and Ca^2^+/calmodulin-dependent protein kinase II (CaMKII) (Taguchi et al., 2007; Serasinghe et al., 2015; Lima et al., 2018). The dynamic balance between phosphorylation and dephosphorylation at Ser616 and Ser637 underpins the stability of mitochondrial dynamics. Further studies are required to gain a deeper understanding of these interrelationships between different modifications.

Mitochondria interact with various organelles within the cellular milieu, with extensive research focusing on their interactions with the endoplasmic reticulum (ER). Mitochondria-ER contact sites (MERCs) are the points of contact between the ER and OMM (Janikiewicz et al., 2018; Magalhães Rebelo et al., 2020). It has been observed that mitochondrial fission often occurs near these MERCs (Friedman et al., 2011), and the replication of mtDNA may also influence the fission site, potentially correlating with fission signals (Murley et al., 2013; Lewis et al., 2016). Upon initiation of fission signals, the concentration of Ca^2+^ in the ER increases, and the ER encircles the mitochondria for an extended period. During this time, cytosolic actin, along with ER-localized inverted formin 2 (INF2) and Spire1C on the OMM, self-assembles into fibers. With the assistance of myosin IIA, this leads to the generation of constrictive force, facilitating the initial contraction of the mitochondria (Ramabhadran et al., 2013; Sun et al., 2013; Gurel et al., 2015). Subsequently, Ca^2+^ permeates from the ER to the mitochondria through the inositol trisphosphate receptor (iP3r)–GrP75–voltage-dependent anion channel (VDAC) complex located within the MERCs, promoting the contraction and fission of the IMM (Szabadkai et al., 2006). This process occurs independently of Drp1 (Chakrabarti et al., 2018). Recent research suggests that endophilin B1 also participates in IMM fission by binding to prohibitin 2 on the IMM, thus facilitating fission (Cho et al., 2019b). The aggregation of actin is crucial for maintaining the activity of Drp1 on the OMM; inhibiting actin aggregation leads to a reduction in Drp1 accumulation and, consequently, mitochondrial fission (Ji et al., 2015).

In addition, the recruitment of Drp1 to the fission site relies on adaptors that facilitate its binding to the OMM and enable its fission function. In mammals, four adaptors are believed to be involved: fission 1 (Fis1), mitochondrial fission factor (Mff), and mitochondrial division (MiD) 49 and 51. While Fis1 plays a significant role in yeast mitochondrial fission, its function in mammals remains unclear. Recent research suggests that Fis1 may primarily facilitate mitophagy or apoptosis (Kim et al., 2011; Qi et al., 2013). Furthermore, it has been reported that Fis1-mediated fission may not be directly linked to Drp1 but achieved through the inhibition of fusion protein GTPase activity (Yu et al., 2019a). Mff plays pivotal in the recruitment of Drp1, and its absence often leads to more severe phenotypes of mitochondrial fission defects compared to the other three adaptors. However, the activation mechanism of Mff on Drp1 remains unclear. Research suggests that due to the variable domain of Drp1, Mff alone is inadequate for binding and activating Drp1 (Liu and Chan, 2015). Furthermore, co-immunoprecipitation experiments have not revealed a direct interaction between Mff and Drp1, likely due to Mff’s low affinity (Clinton et al., 2016), indicating that alternative mechanisms are involved in Mff’s recruitment and activation of Drp1. Recent research proposes a possible mechanism by which Mff recruits Drp1, suggesting that Mff activates Drp1 through its oligomerization (Liu et al., 2021). Similar to Mff and Fis, MiDs can activate Drp1, which promotes its recruitment to the OMM, and thereby enhancing mitochondrial fission (Atkins et al., 2016). When Drp1 is recruited to the mitochondrial fission site, it undergoes structural changes, causing the constriction of mitochondrial tubules and triggering fission events. Some studies suggest that dynamin 2 (DNM2) is responsible for the final membrane cutting in Drp1-mediated mitochondrial fission (Lee et al., 2016). However, this assertion has been challenged by experiments involving DNM2 knockdown and DNM1, DNM2, and DNM3 triple-knockout mouse fibroblasts, which did not exhibit defects in mitochondrial fission (Fonseca et al., 2019). Consequently, mitochondrial fission is a complex process that requires further elucidation through continued research.

### 2.2 Mitochondrial fusion

Mitochondrial fusion involves the merging of the OMM and the IMM, with IMM fusion occurring shortly after OMM fusion. In mammals, Mitofusins1 (Mfn1) and Mitofusins2 (Mfn2) are responsible for OMM fusion, while Optic Atrophy 1 (Opa1) oversees IMM fusion (Chan, 2006; Song et al., 2009). All three proteins belong to the dynamin superfamily and possess GTPase activity (Praefcke and McMahon, 2004). The process of mitochondrial fusion is complex, and we will first discuss the fusion mechanism of the OMM.

Once the two opposing OMM approach each other and interact, the process of fusion is initiated. The formation of homotypic and heterotypic trans-dimers of Mfn1/Mfn2 is essential for tethering and pulling together two mitochondria, enabling fusion to occur (Koshiba et al., 2004; Qi et al., 2016; Li et al., 2019). Traditionally, the structure of Mfn1 has been divided into four domains: the N-terminal GTPase domain, two heptad repeats (HR1, HR2), and the transmembrane (TM) region located between HR1 and HR2 (Koshiba et al., 2004). Bacterial Dynamin-Like Proteins (BDLP) are dynamin-like proteins found in bacteria that are functionally similar to dynamin proteins in animal cells and mediate bacterial membrane fusion. The spatial structure of BDLP has been well-characterized (Low et al., 2009; Bürmann et al., 2011; Bramkamp, 2012). Due to the similarities in structure and biological processes between BDLP and Mfns, researchers have recently redefined the structure of Mfn1 (Qi et al., 2016; Cao et al., 2017; Yan et al., 2018; Li et al., 2019). In addition to the classic GTPase domain, a four-helix bundle known as the HB1 domain (also called HD1) has been identified. The HB1 domain, along with the G domain, which is adjacent to it, is collectively referred to as the minimal GTPase domain (MGD). The structure of HB2 (also called HD2), the transmembrane segments (TMs), and the cytosolic tail (CT) have been predicted based on the researchers understanding of BDLP’s structure. Among these domains, the G domain in the MGD is crucial for the formation of Mfn1 dimers. The GTPase domain consists of an eight-stranded β-sheet surrounded by eight α-helices. In the nucleotide-free (apo) state, the nucleotide-binding pocket is occupied by the large side chain of Trp239. The binding of GTP causes Trp239 to move away, allowing the proper positioning of Asn237 and Asp240 to dock the guanine base, a process referred to as the tryptophan switch. Mutation of the tryptophan residue impairs the nucleotide-binding ability and GTPase activity of Mfn1, preventing the formation of dimers. Furthermore, the mutation of other amino acid residues in the G interface affects the formation of Mfn1 dimers to varying degrees, underscoring the importance of the G domain (Cao et al., 2017). Recent research suggests that the nucleotide binding and dimerization of Mfn1 may require the participation of potassium ions (Yan et al., 2018). The binding of K^+^ and Mg^2+^ to the nucleotide pocket enhances its selectivity, further highlighting the importance of the G domain in Mfn1 dimerization.

Upon mitochondrial collision, Mfn1 on the opposing OMM forms dimers by binding GTP, establishing a trans-linkage. Following dimer formation, two HB1 domains can either orient in opposing directions, referred to as “HB-open,” or in the same parallel direction, known as “HB-closed.” Initially, when the distance between two mitochondria is substantial, HB1 assumes an open state upon dimer formation. Subsequent GTP hydrolysis induces a conformational change in HB1, shifting it to a closed state. This conformational change draws the OMM closer, promoting fusion (Yan et al., 2018). Mfn2 exhibits a similar structure to Mfn1 with 80% sequence similarity (Chen et al., 2003). Reports suggest that heterodimer trans-linkages between Mfn2 and Mfn1, facilitated by the G domain, promote OMM fusion (Li et al., 2019). Furthermore, heterodimers formed by Mfn1/Mfn2 exhibit greater fusion efficiency than homodimers in mouse embryonic fibroblasts (Hoppins et al., 2011). Thus, Mfn1 and Mfn2 play essential roles in OMM fusion.

Unlike Mfns-mediated fusion, which requires trans-linkage formation, OPA1 can facilitate IMM fusion by simply being present on only one IMM of the fusing mitochondria (Mishra et al., 2014). In human cells, OPA1 exists in eight splice forms collectively known as long forms of OPA1 (L-OPA1), all containing an S1 protease cleavage site. The OMA1 protease can cleave L-OPA1 at the S1 site, producing short forms of OPA1 (S-OPA1). Four splice forms possess an S2 cleavage site, which can be cleaved by YME1L1 to generate other S-OPA1 forms (Delettre et al., 2001; Anand et al., 2014). Additionally, the presenilin-associated rhomboid-like protein (PARL) may participate in the generation of S-OPA1 (Cipolat et al., 2006; Saita et al., 2017). Therefore, OPA1 exhibits various forms within the cell.

**FIGURE 1:**
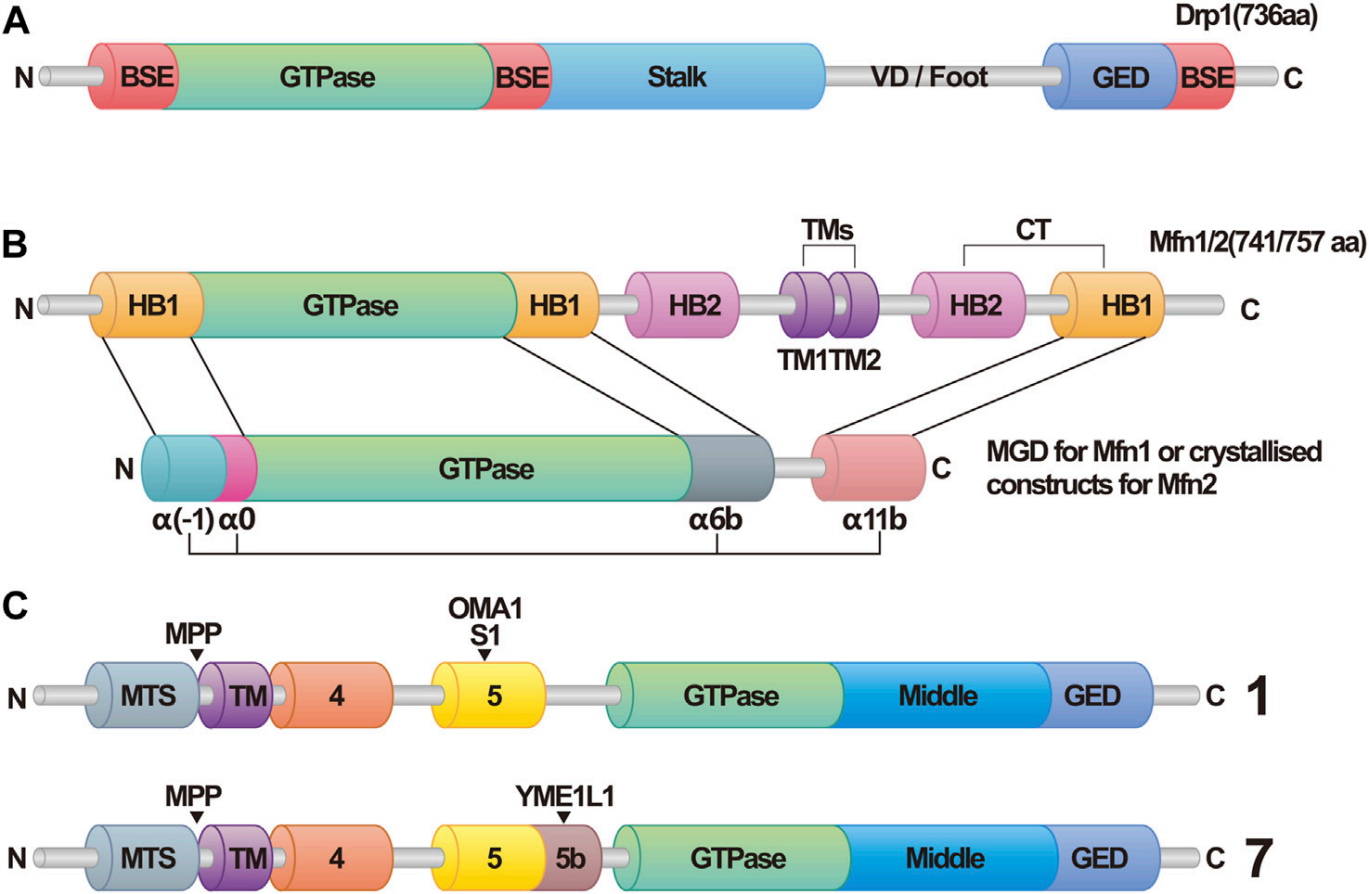
Structural domains of dynamin related protein 1 (Drp1), optic atrophy 1 (OPA1), and mitofusins 1/2 (Mfn1/2). **(A)** Schematic Diagram of Drp1. Abbreviations: Drp1, dynamin related protein 1; BSE, bundle signaling element; VD, variable domain; GED, GTPase effector domain. **(B)** Schematic diagram of Mfn1/2. In this structure, helix bundle 1 (HB1) forms a four-helix bundle that, along with the GTPase in Mfn1/2, makes up the minimal GTPase domain (MGD). The four α-helices are identified as α(-1), α0, α6b, and α11b. In contrasts, this structure in Mfn2 is referred to as crystallized constructs, and has the four α-helices labeled as α1^H^, α2^H^, α3^H^, and α4^H^. Abbreviations: Mfn1/2, mitofusins 1/2; HB1/2, helix bundle 1/2; TMs, transmembrane segments; MGD, minimal GTPase domain. **(C)** Schematic Diagram of OPA1. OPA1 has eight types of RNA splice forms. All OPA1 variants are cleaved at the mitochondrial targeting sequence (MTS) by the matrix processing protease (MPP) once it enters the mitochondria, leading to the generation of long forms of OPA1. Displayed here are Isoform 1 and Isoform 7. Exon 5 in Isoform 1 contains the S1 cleavage site that can be hydrolyzed by OMA1, whereas Exon 5b in Isoform 7 includes the S2 cleavage site, which can be hydrolyzed by Yme1L1, resulting in various forms of short isoforms. Abbreviations: MTS, mitochondrial targeting sequence; MPP, matrix processing protease.

**FIGURE 2:**
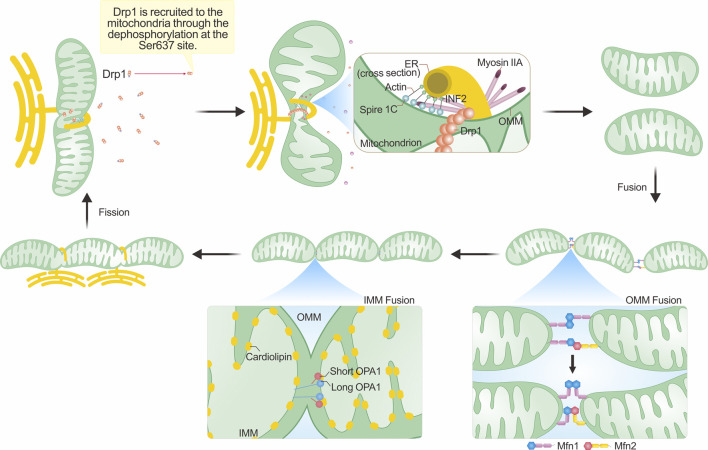
Mechanisms underlying mitochondrial morphological changes. The integral process of mitochondrial fission hinges on the dephosphorylation of Drp1 and its subsequent enlistment to the outer mitochondrial membrane (OMM). This recruitment commonly occurs close to the mitochondria-ER contact sites (MERCs), with actin assembly assuming a crucial role. The polymerization of actin, in tandem with the contraction forces generated with the assistance of myosin IIA, results in initial mitochondrial constriction. Such a process reduces the mitochondrial tubule to a size amenable for inclusion within the Drp1 ring formation. The succeeding hydrolysis of GTP by Drp1 then catalyzes mitochondrial fission. The process of mitochondrial fusion involves the fusion of both the OMM and the inner mitochondrial membrane (IMM). The fusion of the outer membrane is primarily facilitated by Mfn1/Mfn2. In this figure, we employ the single transmembrane model of Mfns to illustrate their topological organization (Mattie et al., 2018). These proteins generate either homotypic or heterotypic dimers that ease OMM fusion via 'HB-open' and 'HB-closed' transformations. In the IMM, the interplay between L-OPA1 and cardiolipin located on the opposing IMM augments IMM fusion, an enhancement further amplified by S-OPA1. For an exhaustive explanation, please refer to the text section titled “Mechanisms underpinning mitochondrial morphological alterations.”

During IMM fusion, L-OPA1 anchors onto the IMM and interacts with cardiolipin (CL) on the opposing IMM. GTP hydrolysis facilitates this interaction, promoting IMM fusion, a process expedited by S-OPA1(Ban et al., 2017). However, when only L-OPA1/S-OPA1 is present, mitochondrial fusion is limited. Hence, maintaining an appropriate ratio of L-OPA1 to S-OPA1 is vital for IMM fusion in cells (Del Dotto et al., 2018). Furthermore, OPA1 is essential for maintaining the morphology of mitochondrial cristae, which is the site of oxidative phosphorylation in the respiratory chain (Frezza et al., 2006). Consequently, OPA1 plays a significant role in IMM fusion and overall mitochondrial function. Figure 1 depicts the structural domains of Drp1, OPA1, and Mfn1/2, while Figure 2 vividly illustrates the fundamental processes associated with mitochondrial fission and fusion. These mitochondrial dynamics play a crucial role in various cellular functions.

## 3 Mitochondrial dynamics and cellular functions

The kidney, an essential organ in the human body, plays a pivotal role in maintaining the stability of the internal environment. It carries out key functions such as waste and toxin elimination, regulation of water and electrolyte balance, maintenance of acid-base balance, and blood pressure regulation. These functions are primarily executed by the nephron, the fundamental unit of the kidney, which ensures normal kidney function. As previously mentioned, the kidney is a mitochondria-rich organ (Bhargava and Schnellmann, 2017), but the distribution of these organelles varies across different segments of the nephron. Studies have documented that the proximal and distal tubules have a high mitochondrial density (Subramanya and Ellison, 2014; McCormick and Ellison, 2015; Molitoris et al., 2022). In contrast, podocytes exhibit notably lower mitochondrial density (Brinkkoetter et al., 2019), a trend also observed in the collecting ducts (Pfaller and Rittinger, 1980; Ahmad et al., 2021). A high mitochondrial density in cells suggests elevated energy consumption, necessitating a substantial oxygen supply. These cells, particularly within renal tubules, are susceptible to changes in oxygen pressure and are prone to injury from various external stimuli leading to decreased oxygen supply. However, cell sensitivity to insults is not solely influenced by high energy consumption, as indicated by mitochondrial density. Other factors, such as local blood supply conditions and capacity for anaerobic glycolysis, also play crucial roles. Studies indicate that the proximal tubular S_3_ segment and the medullary thick ascending limb (mTAL) are the two sections most susceptible to insults (Harvig et al., 1980; Lankadeva et al., 2019; Heyman et al., 2020). These regions exhibit characteristics of high energy consumption and poor blood supply. Notably, the S_3_ segment also has limited capabilities for anaerobic glycolysis. Despite mTAL cells having a considerable capacity for anaerobic metabolism, their high metabolic rate limits their survival under conditions of constrained oxygen supply (Scholz et al., 2021). Therefore, the sensitivity of cells in various nephron segments to external stimuli is multifaceted, underlining the need for further comprehensive research. The nephron is responsible for filtration, secretion, and reabsorption, with the renal tubule, particularly the primary renal tubular epithelial cells (RPTCs), facilitating secretion and reabsorption. RPTCs account for over two-thirds of filtrate reabsorption and complete reabsorption of nutrients like glucose and amino acids (Curthoys and Moe, 2014). Consequently, RPTCs require a significant amount of ATP to support substance transportation, making them one of the cell types with the highest mitochondrial content in the body. These cells are also highly susceptible to injury (Qiu et al., 2018), underscoring the critical role of mitochondria in maintaining tubular and kidney function. Mitochondrial dynamics, as one of the fundamental regulatory mechanisms of mitochondrial function, not only influence cellular energy metabolism but also modulate apoptosis, levels of reactive oxygen species (ROS), mitophagy, and ion metabolism, thereby affecting normal cellular functions. In this section, we summarize and discuss research on the role of mitochondrial dynamics in the regulatory mechanisms of cellular functions.

### 3.1 Mitochondrial dynamics and ATP

Mitochondria primarily generate ATP through oxidative phosphorylation, a process that occurs in the respiratory chain complexes located at the folds of the mitochondrial inner membrane known as mitochondrial cristae (Cogliati et al., 2013). The morphology of mitochondria, regulated by fission and fusion, can influence the number of cristae and thereby modulate intracellular energy metabolism. When cells experience energy deficiency, intracellular AMP levels rise, activating Protein Kinase A (PKA). PKA phosphorylates Drp1 at the Ser637 position, inhibiting its GTPase activity and preventing fission. This indirectly promotes mitochondrial fusion and increases the number of mitochondrial cristae, thereby maintaining the energy supply during cellular starvation (Gomes et al., 2011). As an energy sensor, the AMP-activated protein kinase (AMPK) plays a vital role in maintaining cellular energy metabolism and is activated during cellular energy depletion (Carling, 2017). Recent studies have demonstrated that the use of 5-Aminoimidazole-4-carboxamide ribonucleoside (AICAR) and Irisin can activate AMPK, leading to a decrease in the expression of Drp1, inhibition of Drp616 phosphorylation, and enhancement of Drp637 phosphorylation. As a result, the recruitment of Drp1 to mitochondria is decreased, leading to a decrease in mitochondrial fission and an increase in ATP production. These findings highlight the crucial regulatory mechanism of AMPK on Drp1 (Wang et al., 2022b; Du et al., 2022; Zhou et al., 2022). Interestingly, it seems that Drp1 might exert control over mitochondrial respiration without directly affecting mitochondrial division. Researchers discovered this by employing various acute methods of Drp1 inhibition, and they observed that changes in mitochondrial morphology were either minimal or non-existent. However, mitochondrial respiration in mouse cardiomyocytes was significantly suppressed. In addition, the use of 15-oxospiramilactone (S3) to induce mitochondrial fusion did not affect the rate of mitochondrial oxygen consumption, suggesting that Drp1 might regulate respiration in a manner not dependent on mitochondrial division, thus influencing ATP levels. Subsequent investigation into the mechanism unveiled that inhibiting Drp1 leads to a reduction in the activity of the electron transport chain. This occurs by diminishing the transient opening of the mitochondrial permeability transition pore (mPTP), resulting in the suppression of mitochondrial respiration levels. This is corroborated by the fact that the respiratory inhibitory effect of Drp1 was eliminated when the mPTP regulatory factor, cyclophilin D, was knocked out. This further demonstrates that the regulation of respiration by Drp1 exists independently of mitochondrial division (Zhang et al., 2017). In this experiment, the authors demonstrated the intricate and diverse functions of dynamins, highlighting the presence of regulatory roles that are independent of mitochondrial dynamics. Furthermore, OPA1, an IMM fusion protein, plays an essential role in maintaining the stability of mitochondrial cristae structure (Lee et al., 2017). It is believed that the assembly of OPA1 forms a left-handed helical complex, which ensures the stability of the cristae structure by constricting the membrane tubes located at the junction of mitochondrial cristae. This mechanism helps to preserve normal oxidative phosphorylation levels (Faelber et al., 2019). The Mitochondrial contact site and cristae organizing system (MICOS) is a large protein complex that aids in the formation of contact points between the IMM and OMM. MICOS also stabilizes membrane curvature to maintain the normal morphological structure of mitochondrial cristae (Rampelt et al., 2017). MIC60, a core protein of MICOS, has been shown to physically interact with OPA1, contributing to the stability of the cristae structure (Glytsou et al., 2016). However, a contradictory study suggested that although OPA1 can interact with MIC60, this interaction is not essential for the formation of Crista Junction (CJ) (Barrera et al., 2016). Thus, further research is needed to reconcile this contradiction.

Sirtuins represent a class of nicotinamide adenine dinucleotide (NAD^+^)-dependent protein deacetylases, involved in many physiological processes including DNA repair, gene transcription, and cellular stress responses. Regarding energy metabolism, sirtuins regulate numerous metabolic enzymes through their deacetylase activity, influencing processes such as fatty acid oxidation, ketone body formation, gluconeogenesis, and amino acid metabolism. Mammals have seven types of sirtuin proteins, from SIRT1 to SIRT7 (Huang et al., 2022). SIRT3, located in the mitochondrial matrix, functions as the primary mitochondrial deacetylase, which influence the mitochondrial energy metabolism. It fine-tunes the respiratory chain complex activity, participates in the tricarboxylic acid cycle, and β-oxidation, suggesting that it strongly affects mitochondrial dynamics (Morigi et al., 2018). An increase in SIRT3 enhances OPA1 activity and inhibits Drp1 recruitment, thereby promoting mitochondrial fusion and augmenting ATP production (Huang et al., 2019). The specific regulatory mechanism is discussed in the fourth section of this article.

NAD^+^ is a crucial cofactor involved in numerous physiological processes. It modulates cellular respiration and energy metabolism. Specifically, in oxidative-reduction reactions, NAD^+^ serves as an electron carrier facilitating these reactions, and a decrease in NAD^+^ levels results in disruption of energy production (Ralto et al., 2020). A recent study established an important connection between mitochondrial dynamics and NAD^+^. Upon OPA1 knockout in tumor cells, the researchers observed damage to mitochondrial cristae and a substantial reduction in the NAD^+^/NADH ratio resulting from OPA1 depletion. This impairment in NADH oxidation subsequently impacts NAD^+^ levels. The hypothesized mechanism is that OPA1 knockout may inhibit mitochondrial fusion, mitochondrial structural integrity, and mitochondrial respiratory chain function, thus indirectly diminishing the level of NAD^+^ (Sessions et al., 2022). However, the study primarily centered on tumor cells, and its relevance to other cell types and disease models requires further investigation.

### 3.2 Mitochondrial dynamics and apoptosis

Two decades ago, studies observed the co-localization of Drp1 and Bax (a pro-apoptotic protein) on mitochondria during cellular apoptosis, suggesting a potential connection between mitochondrial fission/fusion and cell apoptosis (Karbowski et al., 2002). Simultaneous studies demonstrated that inhibiting Drp1 prevented cell apoptosis and mitochondrial outer membrane permeabilization (MOMP) (Frank et al., 2001; Tanaka and Youle, 2008). However, the specific regulatory relationship between Drp1 and Bax remained unclear, raising questions about the role of Drp1 in cell apoptosis. Several studies indicated that Drp1 inhibition did not suppress Bax/Bak-induced cell apoptosis or completely prevent the release of cytochrome c (Parone et al., 2006; Estaquier and Arnoult, 2007), suggesting a potentially more complex regulatory mechanism between Drp1 and Bax. Over the years, a better understanding of the relationship between Drp1 and cell apoptosis has emerged. One hypothesis proposes that Drp1 may induce Bax protein aggregation on the membrane by altering the physical state of the cell membrane (hemifusion/hemifission), thereby promoting MOMP and apoptosis. Additionally, in liposome assays, cytochrome c could trigger membrane hemifusion at pH 6, leading to Bax aggregation. This suggests that the membrane status promotes Bax aggregation, rather than a direct regulatory role of Drp1 on Bax aggregation, as no interaction between Drp1 and Bax was observed (Montessuit et al., 2010). Interestingly, a recent study challenged the absence of interaction between Drp1 and Bax (Jenner et al., 2022). The authors convincingly demonstrated the binding of Drp1 and Bax both *in vivo* and *in vitro* using various techniques such as SMLM, ddFP, FCCS, Peptide Array, and Cross-linking Mass Spectrometry. However, this binding requires the presence of a lipid membrane, which could explain why Montessuit et al. did not observe a direct binding between the two. The authors also confirmed that the binding of Drp1 and Bax promoted Bax activation and pore activity, reaffirming the significance of Drp1 in apoptosis. On the other hand, the mitochondrial anchored RING-finger-containing protein, MAPL, promotes Drp1 SUMOylation through its E3 ligase activity. This stabilizes the contact points between mitochondria and the endoplasmic reticulum, facilitating calcium ion flow into mitochondria, deconstructing OPA1 oligomers, remodelling mitochondrial cristae, and effectively releasing cytochrome c to induce apoptosis (Prudent et al., 2015). In conclusion, the relationship between mitochondrial dynamics and cell apoptosis is exceedingly complex and requires further research for a comprehensive understanding.

### 3.3 Mitochondrial dynamics and oxidative stress

Mitochondrial fission and fusion are closely related to intracellular redox levels, as reactive oxygen species (ROS) are primarily produced due to electron leakage from the mitochondrial respiratory chain. The excessive accumulation of ROS leads to an imbalance in oxidative/reductive states, resulting in oxidative stress. This can cause damage to proteins, lipids, and DNA, leading to cellular dysfunction and potentially cell death (Turrens, 2003). Numerous empirical studies have established a connection between ROS levels and mitochondrial fusion/fission. For instance, a study on fruit fly wound healing found that elevated intracellular ROS levels stimulate Drp1 activation, leading to mitochondrial fragmentation. Conversely, lower ROS levels favor mitochondrial fusion (Muliyil and Narasimha, 2014). A similar observation was made in fibroblasts, where the addition of H_2_O_2_ ubiquitinates Mfn1/Mfn2, promoting mitochondrial fission (Rakovic et al., 2011). In contrast, the addition of the antioxidant Trolox reduces ROS levels, mitigating Mfn2 ubiquitination and promoting mitochondrial filament formation (Distelmaier et al., 2012; Blanchet et al., 2015). Studies suggest that the over-accumulation of ROS-promoting mitochondrial fission may be facilitated through the phosphorylation of the Drp1 Ser616 site, facilitating its translocation to the mitochondrial (Chang et al., 2023).

Furthermore, excessive mitochondrial fission also leads to increased intracellular ROS (Gibellini et al., 2015; Areti et al., 2016). The increase in ROS mediated by Drp1 through at least two pathways: 1) Drp1-induced mitochondrial rupture damages the oxidative respiratory chain, resulting in electron leakage, which combines with oxygen to form ROS; 2) Interaction between Drp1 and Bax enhances pore activity, promoting the occurrence of the mitochondrial permeability transition pore (mPTP), which is associated with elevated ROS levels (Andrienko et al., 2017). However, these mechanisms still require further experimental verification.

Reactive Nitrogen Species (RNS), along with ROS, are reactive molecules within cells and include nitric oxide (NO·), peroxynitrite (ONOO-), and other nitric oxide-derived compounds. Their excessive accumulation leads to oxidative stress and cellular damage. RNS can alter protein activity by nitrating amino acid residues of proteins (Olas and Wachowicz, 2007). It has been demonstrated that during cellular oxidative stress, NO can increase Drp1’s GTPase activity through Cys644S-nitrosylation, thereby promoting mitochondrial fission (Cho et al., 2009). Moreover, NO can enhance Drp1Ser616 phosphorylation, augmenting its recruitment to the mitochondrial outer membrane (Bossy et al., 2010). OPA1 can also undergo S-nitrosylation, but the role of this modification remains unknown, necessitating further research for clarification.

### 3.4 Mitochondrial dynamics and mitophagy

Mitophagy, the process by which damaged mitochondria are segregated, excised, and sent to lysosomes for degradation, is a crucial component of the mitochondrial quality control system. It plays a vital role in maintaining mitochondrial health and ensuring cellular energy supply (Onishi et al., 2021). Numerous studies have demonstrated the link between mitochondrial dynamics and mitophagy. Primarily, the onset of mitophagy involves Drp1-mediated fission of dysfunctional mitochondria, and inhibiting fission often impairs mitophagy (Twig et al., 2008). In the PINK1/Parkin pathway, the subsequent mitophagy process is facilitated by the recruitment of Parkin after PINK1 phosphorylates the Ser228 and Ser402 sites upon stabilization on the OMM, which is closely associated with decreased mitochondrial membrane potential (MMP) (Narendra et al., 2008; Okatsu et al., 2012). Recent studies indicate a correlation between Drp1 and MMP reduction. Once recruited to the mitochondria, Drp1 interacts with Zip1, leading to Zn^2+^ influx and MMP reduction. The decrease in membrane potential can serve as a screening mechanism to identify and mark mitochondria that cannot restore normal MPP, thereby guiding mitophagy (Cho et al., 2019a). Additionally, Drp1-mediated fission prevents abnormal Parkin activation on healthy mitochondria, ensuring precise mitophagy (Burman et al., 2017). However, Drp1 is not mandatory for mitophagy, as certain mitophagy occurrences do not require the presence of Drp1 (Murakawa et al., 2015; Yamashita et al., 2016), highlighting the complexity and diversity of mitophagy. Following PINK1/Parkin activation, ubiquitination of Mfn1/Mfn2 occurs, inhibiting mitochondrial fusion and facilitating mitophagy (Gegg et al., 2010). Moreover, Pink1 can phosphorylate Mfn2, promoting Parkin recruitment and subsequent mitophagy, whereas Mfn1 has been shown to lack Parkin binding activity (Chen and Dorn, 2013). Furthermore, SUMO-specific protease 3 (SENP3) facilitates Fis1 deSUMOylation, promoting deferiprone (DFP)-induced mitophagy.

Multiple Parkin-independent pathways exist in mitophagy. In the FUN14 Domain Containing 1 (Fundc-1) pathway, Fundc-1 interacts with Drp1 and OPA1 to regulate mitophagy activity under different conditions. Under normal circumstances, the interaction with OPA1 is enhanced, inhibiting mitophagy, while mitochondrial stress increases the interaction with Drp1, promoting mitophagy (Chen et al., 2016). Additionally, Bcl2/adenovirus E1B 19-kDa interacting protein 3 (Bnip3) promotes mitophagy by stimulating Drp1 and inhibiting Drp1 reduces mitophagy (Lee et al., 2011).

**FIGURE 3:**
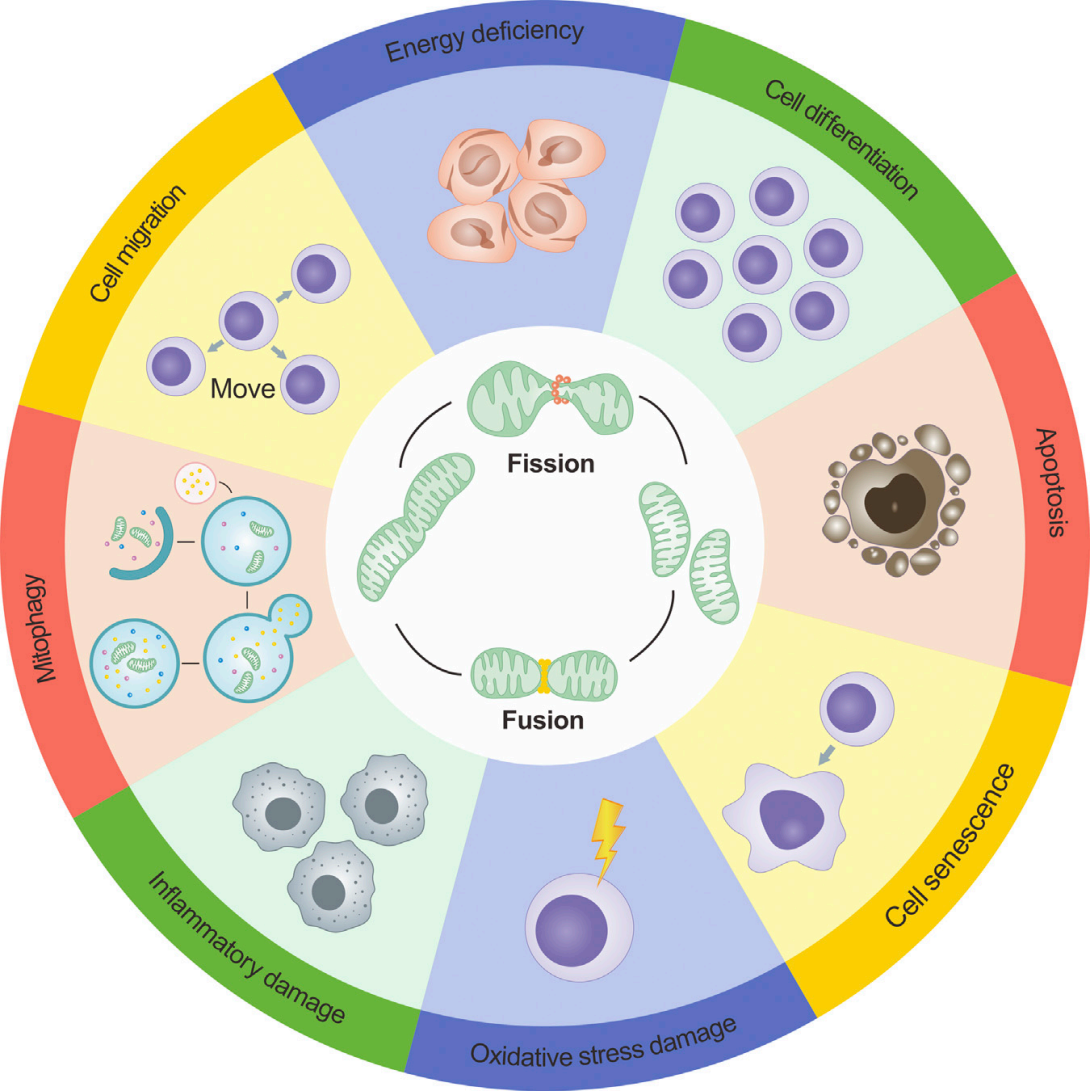
Dynamics of mitochondria and their role in cellular functions. The dynamics of mitochondria are intricately intertwined with cellular operations, and the maintenance of cellular physiological conditions relies on a finely balanced interplay between mitochondrial fission and fusion. However, if the dynamic equilibrium of mitochondrial fission and fusion becomes compromised, it can disrupt cellular functionalities. Such disruptions can precipitate a range of outcomes including decreased energy output, cellular apoptosis initiation, inflammation-induced damage, obstructed mitophagy, and an amplified level of reactive oxygen species (ROS).

Apart from the functions mentioned above, mitochondrial dynamics directly or indirectly affect a range of physiological or pathological processes such as mitochondrial biogenesis, cellular ion exchange, cellular differentiation, cell migration, cellular senescence, and inflammatory changes. This reflects the complex and diverse regulatory roles of mitochondrial dynamics(Dimmer et al., 2008; Dorn et al., 2015; Giacomello et al., 2020; Irazoki et al., 2023). Figure 3 presents a more intuitive representation of the link between mitochondrial dynamics and cellular functions.

## 4 The role of mitochondrial dynamics in acute kidney injury

AKI is a common syndrome with severe clinical outcomes due to decreased kidney function, typically occurring within hours or days. The onset of AKI is characterized by multitude of issues, such as accumulation of metabolic waste in the bloodstream, electrolyte imbalance, decreased urine output or anuria, and fluid overload (Kellum et al., 2021), all of which underscore the importance of early detection and timely intervention. It has been reported that one of the primary sites of injury in AKI is the RPTCs. The AKI damage is accompanied by the disruption of RPTCs brush borders and tight junctions, leading to cell necrosis and shedding, which causes obstruction of the renal tubules, triggering the effusion of filtrate and aggravating the damage (Basile et al., 2012). This suggests that strategies for improving the functionality of RPTCs under various assaults can alleviate AKI. Through the regulation of mitochondrial dynamics and control of mitochondrial function, normal operation of tubular cells can be sustained, presenting a novel approach in AKI treatment.

### 4.1 Mitochondrial morphological characteristics in acute kidney injury

Numerous studies have demonstrated that the mitochondria within RPTCs undergo fragmentation during AKI. Researchers have simulated ischemia-reperfusion (I/R) injury in RPTCs/human kidney proximal tubular epithelial cells (HK-2 cells) using ATP depletion-mediated metabolic stress. Through such models, a noticeable fragmentation of mitochondria has been observed in I/R (Wang et al., 2020a). There have been reports of excessive mitochondrial fragmentation in HK2 I/R models (Ma et al., 2022). Mitochondrial hyper-fragmentation, a distinct feature of AKI, has been observed in several models of AKI, such as, those induced by I/R injury, lipopolysaccharide, cisplatin, and folic acid (Liu et al., 2019a; Aparicio-Trejo et al., 2019; Zhu et al., 2020). This feature, which indicates excessive activation of the mitochondrial fission mediator Drp1, suggests that mitochondrial fragmentation is a major characteristic of AKI onset, and contributes to the death of cells (Frank et al., 2001). Hyper-fragmentation of mitochondria causes a decrease in ATP production, increases levels of ROS, cell apoptosis, and promotes inflammatory responses, all of which enhance the death of RPTCs, thereby aggravating subsequent occurrence of AKI.

The excessive fragmentation of mitochondria observed in AKI can be attributed to specific underlying mechanisms. One such mechanism involves the activation of Drp1, a protein responsible for mediating mitochondrial fragmentation. The key question here is to determine the factors that activate Drp1 in RPTCs under the stress induced by AKI. According to reports, phosphoglycerate mutase family member 5 (PGAM5) may be the cause of Drp1 activation when cells are under stress (Wang et al., 2012). PGAM5, a mitochondrial protein, can be activated by various stress signals and molecules, including ROS, TNF-α, and calcium ionophore. Studies have demonstrated that PGAM5 is upregulated in I/R and cisplatin-induced AKI (Li et al., 2023; Yu et al., 2023), suggesting that it is a possible convergence point of various necrotic pathways. PGAM5L can dephosphorylate Drp1 at Ser 637, thereby activating Drp1’s GTPase activity and increasing mitochondrial fission. Against this background, we infer that the PGAM5/Drp1 signaling pathway may contribute to RPTCs mitochondrial fragmentation during AKI onset. Furthermore, mitochondrial fragmentation leads to the death of RPTCs thus aggravating AKI development.

### 4.2 Mitochondrial dynamics and acute kidney injury recovery

Mitochondrial dynamics not only regulates the progression of AKI, but also in its repair. The prognosis of AKI is dependent on the normal restoration of RPTCs. In the presence of persistent inciting factors, RPTCs undergo maladaptive repair, leading to kidney fibrosis and tubular atrophy, ultimately progressing to chronic kidney disease (CKD) or even end-stage kidney disease (ESKD) (Yu and Bonventre, 2020). This maladaptive repair process in RPTCs is extraordinarily complex, involving multiple factors such as G2/M cell-cycle arrest, cell senescence, profibrogenic cytokine production, and activation of pericytes and interstitial myofibroblasts (Ferenbach and Bonventre, 2015). Recent studies have demonstrated a close relationship between mitochondrial dynamics, AKI recovery, and early fibrosis post-AKI.

In cases of ischemia-reperfusion acute kidney injury (I/R-AKI), researchers have observed that the deletion of Drp1 in proximal tubules reduces kidney damage and fibrosis within the proximal tubule. This preservation of mitochondrial structure and function contributes to the protective effect. It also activates NAD biosynthesis protective to the kidneys, enhancing tubule epithelium proliferation and reducing cell death (Perry et al., 2018). In an AKI-CKD transition model using unilateral ureteral obstruction (UUO), it was reported that UUO-induced renal tubular injury presents more pronounced fibrosis, with mitochondria in UUO kidneys appearing shorter and rounder compared to sham-operated kidneys. Drp1 expression was also elevated in the UUO group, indicating a correlation between mitochondrial fragmentation and kidney fibrosis (Quan et al., 2020).

Additionally, researchers employed a unilateral nephrectomy in combination with contralateral I/R to establish an animal model of AKI progressing to CKD. The findings from this model yielded similar results. Post-IR, mouse kidneys presented fibrosis, significantly reduced Mfn1 expression, and markedly increased Drp1 expression, indicating an imbalance in mitochondrial fusion/fission in the mouse kidneys (Cheng et al., 2022). These experiments suggest that Drp1 may be a robust target for promoting post-AKI recovery and prevent fibrosis.

A recent study showed possible mechanistic insights into Drp1-induced fibrosis post-AKI. This suggests that Drp1 Ser616 phosphorylation-mediated mitochondrial fission might promote fibroblast activation and proliferation through epigenetic regulation mechanisms. Specifically, the phosphorylation of Drp1 at the Ser616 position can acetylate H3K27, an important epigenetic modification. Once H3K27 is acetylated, it can bind to the promoter regions of α-SMA and PCNA, two key genes for fibroblast activation and proliferation, enhancing their transcription and further promoting fibroblast activation and proliferation, leading to kidney fibrosis. This process can be inhibited by mdivi-1 (a Drp1 inhibitor) (Wang et al., 2020c). Nonetheless, the study did not elucidate the reasons behind the phosphorylation of Drp1 at Ser616 and its upstream pathways, indicating the involvement of a complex network in Drp1-promoted fibroblast activation. In conclusion, these studies provide evidence that imbalanced mitochondrial dynamics, leading to mitochondrial fragmentation, have a detrimental impact on AKI recovery. In tubule epithelium cells, mitochondrial fragmentation results in increased cell apoptosis and necrosis, fewer remaining tubule epithelium cells, impeding tubular proliferation and repair. Moreover, mitochondrial fragmentation in renal interstitial fibroblasts leads to fibroblast proliferation and activation, causing kidney fibrosis and accelerating the progression from AKI to CKD.

### 4.3 Targeting mitochondrial fission for the treatment of acute kidney injury

The inhibition of Drp1 activity as a means to prevent excessive mitochondrial fission during AKI has emerged as a promising therapeutic approach. In an I/R model, researchers generated mice with proximal tubule-specific knockout of Drp1. These mice exhibited a decrease in kidney damage induced by I/R, suggesting the potential effectiveness of targeting Drp1 in mitigating AKI. This suggests that strategies that maintain mitochondrial integrity post-Drp1 knockout can resist oxidative stress (Perry et al., 2018). The Regulator of Calcineurin 1 (RCAN1) promotes the recruitment of Drp1 by phosphorylating Mff, leading to mitochondrial division. Excessive cell damage was induced by simulating AKI using I/R and cisplatin, as well as by overexpressing RCAN1 in HK2 cells. Conversely, a lack of RCAN1 resulted in the opposite effect (Xiao et al., 2022). Similarly, like Mff, Fis1 serves as a receptor for recruiting Drp1 to the OMM. Results suggested that the catalytic subunit of DNA-dependent protein kinase (DNA-PKcs) can interact with Fis1, and phosphorylate its Thr34 site, which promotes the binding of Fis1 and Drp1, thereby exacerbating kidney damage induced by AKI (Wang et al., 2022c). It has also been reported that loss of Numb can increase the phosphorylation of Drp1 at the Ser656 site in mice (human Drp1 Ser637 site), thereby promoting mitochondrial recruitment of Drp1. Researchers observed that Numb knockout mice in the renal proximal tubule exhibited more pronounced fragmentation and dysfunction of mitochondria following cisplatin induction compared to normal mice. Additionally, these mice also experienced more severe renal tubular damage. However, application of mdivi-1 significantly improved the kidney function in these mice (Liu et al., 2019b). In addition, suppressing the phosphorylation of Drp1 at the Ser616 site can reduce the recruitment of Drp1 to the OMM, and inhibit excessive mitochondrial division (Chen et al., 2020a). A recent study showed that emodin inhibited the kinase activity of calcium/calmodulin-dependent protein kinase II, preventing its phosphorylation of Drp1 at the Ser616 site to alleviate I/R-induced kidney damage (Wang et al., 2022d).

Inhibiting Drp1 also presents a promising approach for mitigating kidney damage in sepsis-induced AKI (S-AKI). By using lipopolysaccharide (LPS) to establish S-AKI models in animals and cells, and inhibiting Drp1 activity with mdivi-1, it was found that it is possible to reduce the activation of the NLRP3 inflammasome-mediated pyroptosis pathway, thereby improve S-AKI in animals and cells (Liu et al., 2020). SUMO-specific proteases 3 (SENP3), a member of the deSUMOylating enzyme family, promotes the binding of Drp1 and Mff through deSUMOylation of Drp1, leading to an increase in mitochondrial fission (Guo et al., 2017). In the study of S-AKI, researchers investigated the role of SENP3. They found that overexpressing SENP3 in NRK-52E cells, after treatment with LPS, led to a significant increase in cytoplasmic cytochrome c levels and caspase-3 expression compared to the group with SENP3 knockdown. These findings indicated the occurrence of apoptosis. Moreover, there was a further reduction in mitochondrial membrane potential, suggesting the presence of mitochondrial damage. Therefore, inhibiting SENP3 and reducing the recruitment of Drp1 is an effective strategy to alleviate S-AKI (Wang et al., 2022a).

Besides inhibiting the recruitment of Drp1, AKI can also be mitigated by regulating mitophagy through Drp1. As mentioned earlier, Fundc-1 is a Parkin-independent mitophagy pathway that is activated under cellular hypoxic conditions. It can directly bind to microtubule-associated proteins 1A/1B light chain 3B (LC3) to induce mitophagy (Liu et al., 2012). Through the use of ischemic preconditioning techniques, researchers have discovered that it can mitigate kidney damage in mice following I/R injury. However, this protective effect of ischemic preconditioning is hindered when Fundc-1 is knocked out. This suggests that ischemic preconditioning activates the Fundc-1-induced mitophagy pathway, which facilitates the degradation of damaged mitochondria during I/R injury and confers a protective effect. Elsewhere, Fundc-1 knockout resulted in mitochondria over-fission through a mechanisms involving Drp1, leading to the formation of several undegradable mitochondrial fragments, thereby triggering cell apoptosis, damaging the tubular epithelium, and aggravating AKI (Wang et al., 2020b). In a separate experiment involving kidney I/R, researchers discovered that SIRT3, a crucial deacetylase enzyme located within mitochondria, plays a pivotal role in mitigating kidney damage. They observed that SIRT3 enhances mitophagy, a process by which damaged or dysfunctional mitochondria are selectively removed, thereby providing a protective effect against kidney injury. Overexpression of SIRT3 significantly increased mitophagy and reduced the expression level of Drp1. Conversely, the expression of Drp1 reduced the level of mitophagy. They also found that downregulation of SIRT3 expression and upregulation of Drp1 expression caused the most severe mitochondrial damage, i.e., mitochondria were excessively divided, and mitophagy was inhibited in the most severe state. The collective findings from these experiments strongly suggest that SIRT3 plays a role in modulating mitophagy by regulating the Drp1 pathway. This regulatory mechanism appears to have a beneficial effect in alleviating the detrimental impact of kidney I/R injury (Zhao et al., 2021).

In conclusion, the above experiments demonstrate the feasibility of targeting Drp1 for the treatment of AKI. However, Drp1 has various modifications and regulatory methods, indicating that there is significant potential for exploring the therapeutic potential of Drp1.

### 4.4 Targeting mitochondrial fusion for the treatment of acute kidney injury

Promoting mitochondrial fusion as a means to alleviate AKI has gained significant attention among researchers, similar to the concept of inhibiting mitochondrial fission. A crucial player in maintaining the structural integrity of mitochondrial cristae is OPA1, an IMM fusion protein. However, under conditions of mitochondrial stress, OMA1 can become activated, resulting in the cleavage of OPA1. This cleavage event disrupts mitochondrial fusion and compromises the stability of cristae. However, inhibiting OMA1 activity can reduce the hydrolysis of OPA1 during cell stress, thereby promoting mitochondrial fusion and cell survival (Head et al., 2009). In a previous study, a model of I/R was simulated using ATP-depletion injury in RPTCs. Results showed that the hydrolysis of OPA1 was increased. Knock down or knock out of OMA1 in RPTCs and mice, respectively, induced I/R injury, resulting in significantly lower mitochondrial fragmentation, release of cytochrome c, and cell apoptosis (Xiao et al., 2014). In a separate study, researchers investigated the OMA1-OPA1 mechanism and its potential therapeutic implications. They pre-treated RPTCs with hyperoside before inducing the IRI model. The findings revealed that hyperoside effectively inhibited the hydrolysis of OPA1 by OMA1, resulting in the mitigation of AKI. This suggests that targeting the OMA1-OPA1 pathway, such as with the use of hyperoside, holds promise as a therapeutic strategy for AKI (Wu et al., 2019).

Besides OMA1, YME1L1 is another protease that can hydrolyze OPA1. OMA1 cleaves OPA1 at the S1 site, while YME1L1 targets the S2 site. It has been reported that S-OPA1, produced by YME1L1 cleavage of OPA1, promotes mitochondrial fusion (Mishra et al., 2014). In a study investigating S-AKI, researchers used LPS to induce S-AKI in mice and an HK2 cell model. They examined mitochondrial morphology, apoptosis markers, and ROS levels. The results showed that overexpressing SIRT3 significantly alleviated these indicators, including a reduction in fragmented mitochondria. These findings suggest that SIRT3 has a protective effect against S-AKI by mitigating mitochondrial dysfunction and reducing apoptosis and oxidative stress. Through functional experiments, it was confirmed that SIRT3 can deacetylate YME1L1 and promote OPA1-mediated mitochondrial fusion by inhibiting L-OPA1 (Jian et al., 2023). Interestingly, besides deacetylating YME1L1, SIRT3 can directly deacetylate OPA1.

In a study investigating cisplatin-induced AKI, researchers utilized matrine to enhance the deacetylation activity of SIRT3 on OPA1. This intervention resulted in the alleviation of renal dysfunction, histological damage, and inflammation in mice (Yuan et al., 2022). In another experiment aiming to rescue I/R-AKI, researchers observed that overexpressing SIRT3 led to the activation of the ERK signaling pathway. This activation, in turn, enhanced the expression and activity of OPA1, promoting mitochondrial fusion. As a result, the mitochondria were protected from injury caused by ischemia/reperfusion (Wang et al., 2019). Moreover, SIRT3 was found to be a mediator of the ubiquitination and degradation of Mfn2 in other I/R-AKI experiments, thus alleviating AKI (Shen et al., 2021). In conclusion, SIRT3 plays multiple functions in mitochondrial dynamics, which need to be further investigated.

MicroRNAs are a class of small non-coding RNA molecules that primarily regulates gene expression by binding to specific areas of mRNA, thereby inhibiting their translation (Kaucsár et al., 2010). miR-214, a type of microRNA, has been identified to be a direct target of Mfn2. In mice I/R-AKI experiments, miR-214 was increased and overexpression of miR-214 enhanced the mitochondrial fragmentation and increased apoptotic RPTCs. In contrast, when anti-miR-214 was applied, it significantly alleviated these phenotypes. The experimental results confirmed that miR-214 suppressed the expression of Mfn2, which led to the inhibition of mitochondrial fusion and the promotion of mitochondrial division. These effects of miR-214 ultimately contributed to the observed phenotypes (Yan et al., 2020). In a similar study, Mfn1 was found to be a target of miR-125b. In a cisplatin-induced AKI model, it was found that miR-125b inhibited Mfn1 expression, thereby enhancing mitochondrial fission and cell apoptosis. Inhibition of miR-125 expression mitigated both mitochondrial dysfunction and kidney injury (Zhao et al., 2022).

These experiments all demonstrate that the suppression of Mfn1/Mfn2 could lead to mitochondrial dysfunction and exacerbate AKI. However, other scholars have reported opposite results (Gall et al., 2015). Investigators generated conditional knockout (cKO) mice specifically targeting Mfn2 in the proximal tubule (cKO-PT Mfn2) and used an I/R model to assess kidney injury. Based on previous studies, it was initially expected that the absence of Mfn2 would exacerbate kidney damage in the I/R model. However, surprisingly, the survival rate of the Mfn2 knockout mice significantly increased following ischemic injury. Furthermore, there was no significant difference in the degree of cell apoptosis or necrosis 24 h post-ischemia compared to the control group. To explain this anomaly, mechanistic experiments were performed which showed that it was caused by proliferation of renal tubular epithelial cells. Under physiological conditions, Mfn2 can inhibit the Ras/ERK signaling pathway, which is a key pathway regulating cell proliferation (Khavari and Rinn, 2007). Loss of Mfn2 stimulates the Rsa/ERK pathway, thereby increasing the proliferation of renal tubular epithelial cells and alleviating I/R-induced kidney damage (Gall et al., 2015). To provide a concise overview for readers, the key findings and results from the research in this chapter are summarized in Table 1 **.**

**TABLE 1:** Targeting mitochondrial dynamics for the treatment of AKI.

Therapeutics	Research subject	Type of injury	Mechanism	Results	References
Genetic deletion of Drp1 in RPTCs	Mice	I/R-AKI	Loss of Drp1 results in inhibition of mitochondrial fission	Reducing oxidative stress, tubular atrophy, and fibrosis	Perry et al. (2018)
RCAN1	Mice/HK2	I/R-AKI	RCAN1 promotes the recruitment of Drp1 by enhancing the phosphorylation of Mff	The lack of RCAN1 significantly reduces kidney injury, while overexpression of RCAN1 leads to the opposite phenotype	Xiao et al. (2022)
		Cis -AKI			
DNA-PKcs	Mice/HK2	I/R-AKI	DNA-PKcs interacts with Fis1 and promotes the recruitment of Drp1 by phosphorylating at the Thr34 site	Tubule-specific ablation of DNA-PKcs diminishes AKI-mediated renal dysfunction and tubular death	Wang et al. (2022c)
		Cis -AKI			
		S-AKI			
Numb	Mice/NRK-52E	I/R-AKI	Loss of Numb increases the phosphorylation of Drp1 at Ser656 site in mice (Ser637 site in human Drp1)	Tubular-specific deletion of Numb aggravates AKI	Liu et al. (2019b)
		Cis -AKI			
Emodin	Mice/HK2	I/R-AKI	Emodin reduced Drp1 phosphorylation at Ser616	Emodin prevents mitochondrial fission and restores the balance of mitochondrial dynamics	Wang et al. (2022d)
Mdivi-1	Mice/TCMK-1	S-AKI	Mdivi-1 reduces pyroptosis by inhibiting mitochondrial fission and preserving mitochondrial function	Mdivi-1 inhibits Drp1 activity and reduces the activation of the pyroptosis pathway mediated by NLRP3 inflammasome	Liu et al. (2020)
SENP3	Mice/NRK-52E	S-AKI	SENP3 deSUMOylates Drp1 and enhances Drp1 binding to the mitochondrial adaptor protein Mff	SENP3 knockdown significantly improves LPS-induced apoptosis in NRK-52E cells, while SENP3 overexpression aggravates it	Wang et al. (2022a)
Fundc-1	Mice/RPTCs	I/R-AKI	Fundc1 eliminates mitochondrial-localized Drp1 via mitophagy	Fundc1 deficiency leads to kidney injury, severe pro-inflammatory response, and tubular cell death	Wang et al. (2020b)
SIRT3	Mice/NRK-52E	I/R-AKI	SIRT3 mitigates IRI-induced kidney damage by regulating DRP1 and inducing mitophagy	Protects against mitochondrial damage during AKI	Zhao et al. (2021)
OMA1	Mice/RPTCs	I/R-AKI	Inhibiting OMA1 activity can reduce the hydrolysis of OPA1, leading to less mitochondrial fragmentation	OMA1 knockout protects renal function and reduces OPA1 proteolysis in ischemic AKI	Xiao et al. (2014)
Hyperoside	Mice/HK2	I/R-AKI	Hyperoside inhibits OMA1-mediated hydrolysis of OPA1, suppressing mitochondrial division	Hyperoside inhibits IR-induced oxidative stress, finally suppressing cell apoptosis	Wu et al. (2019)
SIRT3	Mice/HK2	S-AKI	SIRT3 deacetylates YME1L1 to promote mitochondrial fusion mediated by OPA1 by inhibiting L-OPA1 processing	Overexpression of Sirt3 alleviates LPS-induced apoptosis and mitochondrial injury	Jian et al. (2023)
Matrine	Mice/HK2	Cis -AKI	Matrine enhances the deacetylation of OPA1 by SIRT3	Matrine alleviates renal dysfunction, histological damage, and inflammation	Yuan et al. (2022)
SIRT3	Mice/LLC-PK1	I/R-AKI	SIRT3 overexpression activates the ERK signaling pathway, enhances OPA1 expression, and promotes mitochondrial fusion	Sirt3 overexpression attenuates mitochondrial apoptosis and renal injury	Wang et al. (2019)
SIRT3	Mice/HK2	I/R-AKI	Downregulation of SIRT3 leads to Mfn2 ubiquitination and degradation	Mfn2 ubiquitination and degradation mediate IR-induced AKI, leading to increased apoptosis, oxidative damage, and inflammation	Shen et al. (2021)
miR-214	Mice/RPTCs	I/R-AKI	miR-214 inhibits Mfn2 expression, suppressing mitochondrial fusion	miR-214 promotes mitochondrial fragmentation and kidney cell death	Yan et al. (2020)
miR-125b	Mice/HK2	Cis -AKI	miR-125b inhibits Mfn1 expression, thereby exacerbating mitochondrial fission	By inhibiting miR-125 expression, both mitochondrial dysfunction and kidney injury are alleviated	Zhao et al. (2022)
Conditional Knockout of Proximal Tubule Mfn 2	Mice/RPTCs	I/R-AKI	Mfn2 deficiency alleviates the inhibition on the Ras/ERK pathway, increasing proliferation of renal tubular epithelial cells	Survival rates are significantly increased in Mfn2 knockout mice after ischemic injury	Gall et al. (2015)

RCAN1: Regulator of calcineurin 1; DNA-PKcs: catalytic subunit of DNA-dependent protein kinase; Cis-AKI: cisplatin induced acute kidney injury; S-AKI: sepsis induced acute kidney injury; I/R-AKI: Ischemia-reperfusion induced acute kidney injury; SENP3: SUMO-specific proteases 3; Fundc-1: FUN14 Domain Containing 1; SIRT3: Sirtuin 3; Drp1: dynamin related protein 1; OPA1: optic atrophy 1; Mfn1: mitofusins1; Mfn2: mitofusins2; RPTCs: primary renal tubular epithelial cells; HK2: human kidney proximal tubular epithelial cells.

### 4.5 Exploring small molecule compounds targeting Drp1 and Mfns for the treatment of acute kidney injury

The preceding discussion strongly suggests that intervening in mitochondrial division or promoting mitochondrial fusion presents a promising and novel approach for the management of AKI. As summarized in Table 1, numerous studies have already applied this concept to AKI treatment, leading to the design of a plethora of drugs aimed at targeting mitochondrial dynamics. Mitochondrial division inhibitor-1, or mdivi-1, a member of the quinazolone family of compounds, was one of the earliest discovered inhibitors of mitochondrial division. It inhibits Drp1 GTPase activity and its helical self-assembly to impede mitochondrial division (Cassidy-Stone et al., 2008). Nonetheless, recent research has raised concerns about the specificity of mdivi-1, as it was observed to alleviate oxidative stress through mechanisms unrelated to Drp1 GTPase activity (Duan et al., 2020), and mdivi-1 has shown Drp1-independent utility in several disease models (So et al., 2012; Lucantoni et al., 2018; Ruiz et al., 2018). Results of recent studies have demonstrated that mdivi-1 acts as an inhibitor of Complex I, as it decreases respiration and exhibits relatively low inhibitory effects on Drp1 GTPase activity (Bordt et al., 2017). Despite the mentioned concerns, mdivi-1 has consistently displayed its capacity to effectively preserve mitochondrial morphology and regulate Drp1 phosphorylation in numerous experiments (Zhan et al., 2019; Deng et al., 2020). Although it is not a specific inhibitor of Drp1, it remains a subject of discussion regarding mitochondrial dynamics targeting.

As of now, mdivi-1 has not been utilized in clinical applications. Nevertheless, its promising potential has been demonstrated in various studies, suggesting its efficacy in addressing renal and other systemic diseases, including neurological conditions, cardiovascular, cancer, and metabolic disease domains (Dai et al., 2020; Nhu et al., 2021; Ding et al., 2022; Finocchietto et al., 2022). In addition to inhibiting Drp1 activity, mdivi-1 exerts antioxidative, antiapoptotic, and anti-inflammatory effects (Deng et al., 2020; Bordt et al., 2022; Liu et al., 2022), and thus it can prevent AKI development. Through these effects, mdivi-1 maintains the stability of the mitochondrial network. Multiple AKI models (both in animals and cells) have been used to validate the effects of mdivi-1. In one study related to rhabdomyolysis (RM)-induced AKI, researchers administered mdivi-1 via intraperitoneal injection to rats at 50 mg/kg 1 h prior to RM induction, and then at 6h and 12 h post-induction. They also found that mdivi-1 administration reduced apoptosis of renal tubular cells, mitochondrial division, and ROS levels, thereby alleviating kidney damage (Tang et al., 2013). In another study on contrast-induced AKI (CI-AKI), rats were pretreated with an intraperitoneal injection of mdivi-1 (10 mg/mL, 15 mg/kg) and were then intravenously injected with 15 mL/kg iohexol to induce CI-AKI 2 h later. Cellular experiments involved treating HK2 cells with mdivi-1 (50 μM) for 24 h, followed by the use of iohexol for modeling. Results of the study showed that mdivi-1 pretreatment inhibited mitochondrial division and mitigated oxidative stress, cellular apoptosis, and inflammation, thereby alleviating CI-AKI (Yang et al., 2023). Moreover, as mentioned earlier, mdivi-1 can inhibit the pyroptosis pathway by suppressing mitochondrial division, thereby relieving S-AKI (Liu et al., 2020). In summary, mdivi-1 has showcased its effectiveness across various dimensions and systems, establishing itself as a compound with highly promising research prospects.

P110, a precisely designed selective peptide inhibitor, can specifically block the binding of Drp1 to its mitochondrial ligand Fis1, leading to the inhibition of mitochondrial division without affecting the interaction between Drp1 and Mff or Drp1 and MIEF1. P110 can also inhibit Drp1’s GTPase activity, without inhibiting the GTPase activities of other dynamins such as OPA1, Mfn1, and dynamin 1. A notable feature of P110 is that it only exhibits inhibitory effects on Drp1 under oxidative stress conditions, with minimal impact under basal conditions (Qi et al., 2013). These distinct advantages make P110 a potential drug targeting mitochondrial dynamics. As a novel inhibitor targeting mitochondrial division, P110 can affect various processes in the neurological and cardiovascular systems (Tian et al., 2017; Joshi et al., 2018; Haileselassie et al., 2019; Joshi et al., 2019), but no reports on AKI-related studies exist to date. Therefore, further investigation is needed. Moreover, although P110 has shown considerable therapeutic effects in animal models, its clinical utility is hampered by its vulnerability to degradation by serum and cell proteases, as well as its inability to be administered orally. In the latest study, researchers aimed to overcome these limitations by identifying P110s binding site known as the switch I-adjacent groove (SWAG) and discovering small molecule compounds like SC9 that can effectively bind to this site (Rios et al., 2023). Although SC9 can effectively inhibit mitochondrial division and significantly reduce LPS-induced mortality, it has some drawbacks such as large molecular size (466 Da), high polarity, and a short half-life, necessitating research into SC9 analogues to overcome these limitations.

**FIGURE 4:**
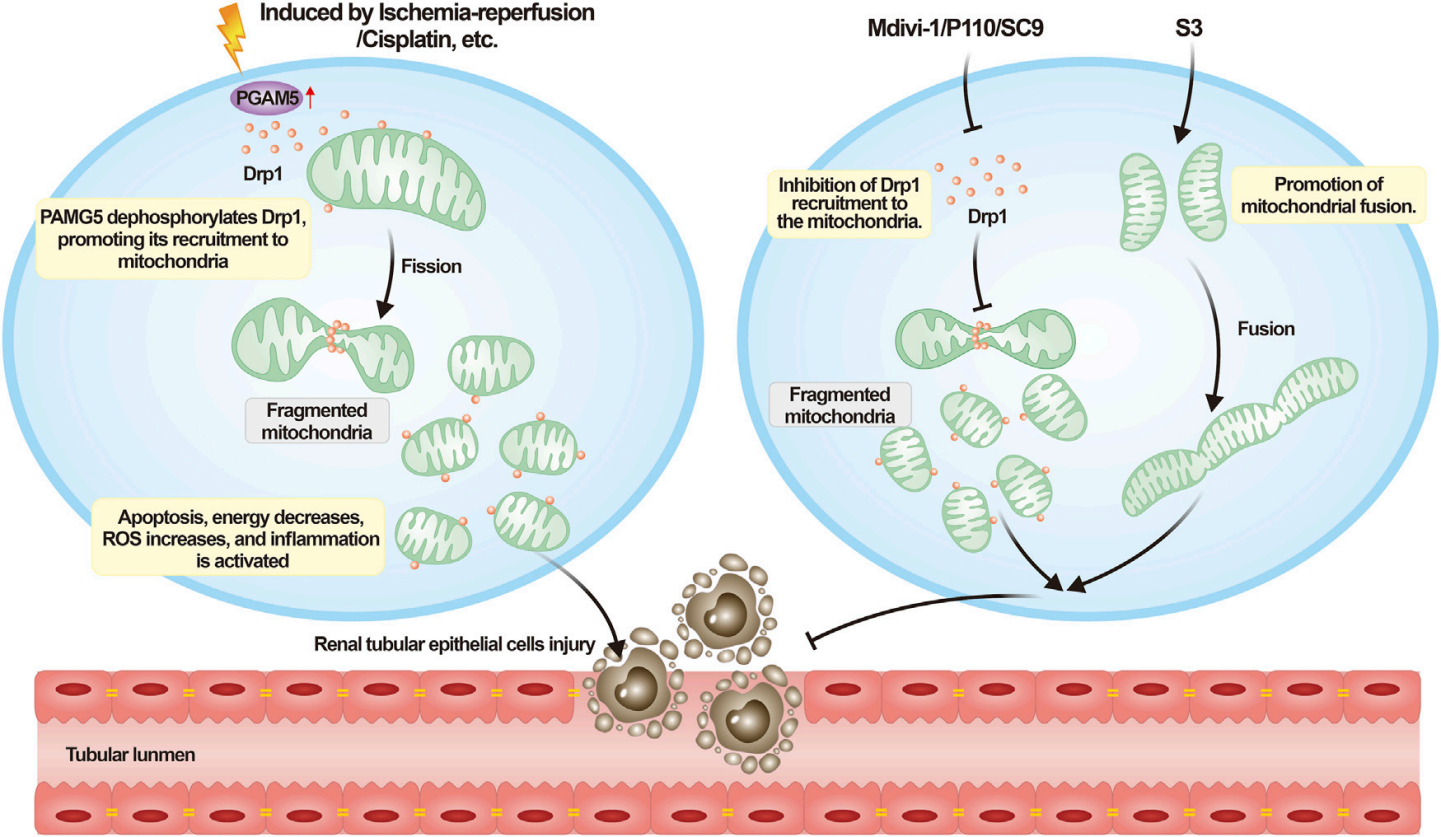
Understanding acute kidney injury from the perspective of mitochondrial dynamics. Upon the onset of AKI, a variety of external necrotic stimuli can affect renal tubular epithelial cells. These necrotic signals activate PGAM5, which dephosphorylates Drp1 Ser 637, consequently enhancing the recruitment of Drp1. This process leads to mitochondrial fragmentation, impairing cellular functionality, triggering cell death, and exacerbating the progression of AKI. Mitochondrial dynamics-targeting compounds such as mdivi-1, P110, and SC9 can inhibit excessive mitochondrial fission, whereas S3 can promote mitochondrial fusion to counteract the aforementioned process.

In addition to the previously mentioned medications, another compound known for promoting mitochondrial fusion is the diterpenoid derivative 15-oxospiramilactone (S3). S3 enhances the activity of Mfns and fosters mitochondrial fusion by inhibiting the deubiquitinase USP30, which augements the non-degradative ubiquitination of Mfns (Yue et al., 2014). Previous research has affirmed the role of S3 in facilitating mitochondrial fusion within murine embryonic fibroblasts (Yue et al., 2014). In a recent study, it was discovered that S3 treatment notably enhances the Parkin-mediated mitophagy pathway, contributing to the elimination of damaged mitochondria and subsequently fostering improved mitochondrial health (Zhuang et al., 2022). Although these compounds demonstrate promising results at the animal level, substantial research is required before their potential clinical application. Figure 4 is utilized as a visual aid to elucidate the process of interpreting AKI in the context of mitochondrial dynamics.

## 5 Conclusion and future outlook

AKI is a severe clinical syndrome with high incidence and mortality rates. Patients with AKI typically show reduced glomerular filtration rate (GFR), increases serum creatinine (SCr), and blood urea nitrogen (BUN), as well as persistent oliguria or anuria. AKI affects the health and quality of life for 10%–15% of hospital patients and over 50% of intensive care unit patients (Ronco et al., 2019). Therefore, early intervention for AKI is of paramount importance to slow disease progression and alleviate kidney damage. Despite the availability of various treatment options for AKI, such as maintaining electrolyte and acid-base balance, avoiding nephrotoxic drugs and therapies, and even resorting to dialysis, these strategies have limitations and are unable to fully reverse the kidney damage caused by AKI, leading to suboptimal outcomes. Hence, effective treatment of AKI remains a significant challenge in clinical medicine, and there is an urgent need to develop more efficient treatment methods and strategies.

Mitochondria, as the powerhouses of the cell, directly influence the survival and function of renal epithelial cells, and thus the overall health of the kidneys, by inducing structural and functional changes. Recent research has revealed the pivotal role of mitochondrial dynamics in the pathogenesis of AKI. During AKI, RPTCs exhibit excessive mitochondrial fission and reduced fusion, i.e., over-activation of Drp1 and over-inhibition of OPA1, Mfn1, Mfn2. Excessive mitochondrial fission often leads to numerous adverse effects, including cell apoptosis, reduced energy output, induction of cell inflammation, and increased oxidative stress, thereby aggravating the course of AKI. Therefore, the inhibition of excessive mitochondrial fission and promotion of mitochondrial fusion have emerged as promising therapeutic strategies for AKI. Multiple studies have provided evidence supporting the feasibility of targeting mitochondrial dynamics as a treatment approach for AKI, which has yielded encouraging therapeutic outcomes. As our understanding of mitochondrial dynamics deepens, we believe we will be capable of developing more efficient AKI treatments, thereby improve patient survival rates and quality of life.
